# Liquid biopsy for monitoring minimal residual disease in localized and locally-advanced non-small cell lung cancer after radical-intent treatment

**DOI:** 10.1016/j.jlb.2024.100145

**Published:** 2024-02-10

**Authors:** Héctor Aguilar, Belén López-Roldán, Anna Vilalta-Lacarra, Gorka Alkorta-Aranburu, R. Claramunt, José Antonio López-Guerrero, S. Sandiego, I. Gil-Bazo

**Affiliations:** a Department of Medical Oncology, Fundación Instituto Valenciano de Oncología, Valencia, Spain; b Department of Medical Oncology, Hospital Santa Bárbara, Soria, Spain; c Department of Medical Oncology, Clínica Universidad de Navarra, Pamplona, Spain; d CIMA LAB Diagnostics, Universidad de Navarra, Pamplona, Spain; e Molecular Biology Laboratory, Fundación Instituto Valenciano de Oncología, Valencia, Spain; f School of Medicine. Universidad Católica de Valencia San Vicente Mártir, Valencia, Spain; g Centro de Investigación Biomédica en Red de Cáncer (CIBERONC), Madrid, Spain

**Keywords:** Liquid biopsy, NSCLC, Minimal residual disease, ctDNA, Epidermal growth factor receptor

## Abstract

Blood-based biomarkers investigation does not require invasive tissue biopsies and may explore diverse tumoral components such as proteins, microRNAs, circulating tumor cells, ctDNA, and exosomes and may better reflect tumor molecular heterogeneity, either temporal or spatial. ctDNA is related to tumor burden and represents a more objective measure of the total body disease burden than imaging findings. ctDNA profiling can be therefore useful to determine minimal residual disease (MRD), which is defined as the remaining tumor cells or tumor-derived material after definitive treatment in patients with no clinical evidence of disease.

The detection of MRD is highly predictive of future disease recurrence. Although detectable MRD is associated with a poor prognosis, it is not clear whether MRD detection can guide therapy escalation to improve patient outcomes.

In this review, we present four cases of epidermal growth factor receptor (*EGFR*) mutant NSCLC patients who received standard of care curative treatment and periodic radiological assessment and liquid biopsy analyses were carried out as follow-up.

A tumor-informed 52 genes Oncomine Pan-Cancer Cell-Free assay (Thermo Fisher Scientific, Waltham, MA, USA), was used to identify single-nucleotide variants (SNVs), indels, copy number variations (CNVs), and RNA fusions in blood based liquid biopsy ctDNA in three of four patients. In one patient the approach used was through Commercial Kit Cobas *EGFR* Mutation Test v2 CE-IVD (Roche Diagnostics SL) that identifies 42 mutations in the *EGFR* gene. In one patient, an actionable oncogene driver alteration was identified in the ctDNA analysis, four months after radical intent concurrent chemoradiotherapy and six weeks before radiological distant relapse was clearly confirmed. There is no evidence of ctDNA or radiological disease relapse in the other three patients.

Finally, a review of the literature addressing the potential value of MRD detection in this clinical setting is presented and discussed as well.

## 1 Introduction

The molecular profiling of solid cancers has been used across multiple cancer types to identify poorer prognosis cancers and guide treatment selection. Most studies have utilized the analysis of DNA and/or RNA and/or protein expression on a tissue biopsy from the primary tumor or less commonly, a metastatic lesion [1].

There are some major limitations with this approach. First, there are practical and clinical challenges to obtain sufficient tissue from poorly accessible metastases and primary samples. Secondly, intra-patient and intra-tumor heterogeneity may result in a missed or incorrectly classified cancer, due to spatial or temporal differences. Third, repeated tumor biopsies to monitor tumor evolution and response dynamics is not clinically feasible, nor ethically acceptable.

Blood-based biomarkers, commonly referred to as liquid biopsies are an alternative or a complement to solid tumor biopsies and imaging studies to better characterize tumor molecular drivers and response to treatment in a non-invasive manner.

The discovery of cell-free DNA (cfDNA) dates back to 1948, when Mandel and Metais found it in the sera of cancer patients [2]. It was first clinically implemented in prenatal diagnostics to detect congenital disorders [3]. In blood, cfDNA is naturally fragmented to a size of 142–170 base pairs and where the fragment sizes relate to the length of DNA wrapped around nucleosomes and the subsequent cleavage of unprotected DNA between nucleosomes by nucleases [4]. Tumor derived cfDNA is also known as circulating tumor DNA (ctDNA).

**Fig. 1:**
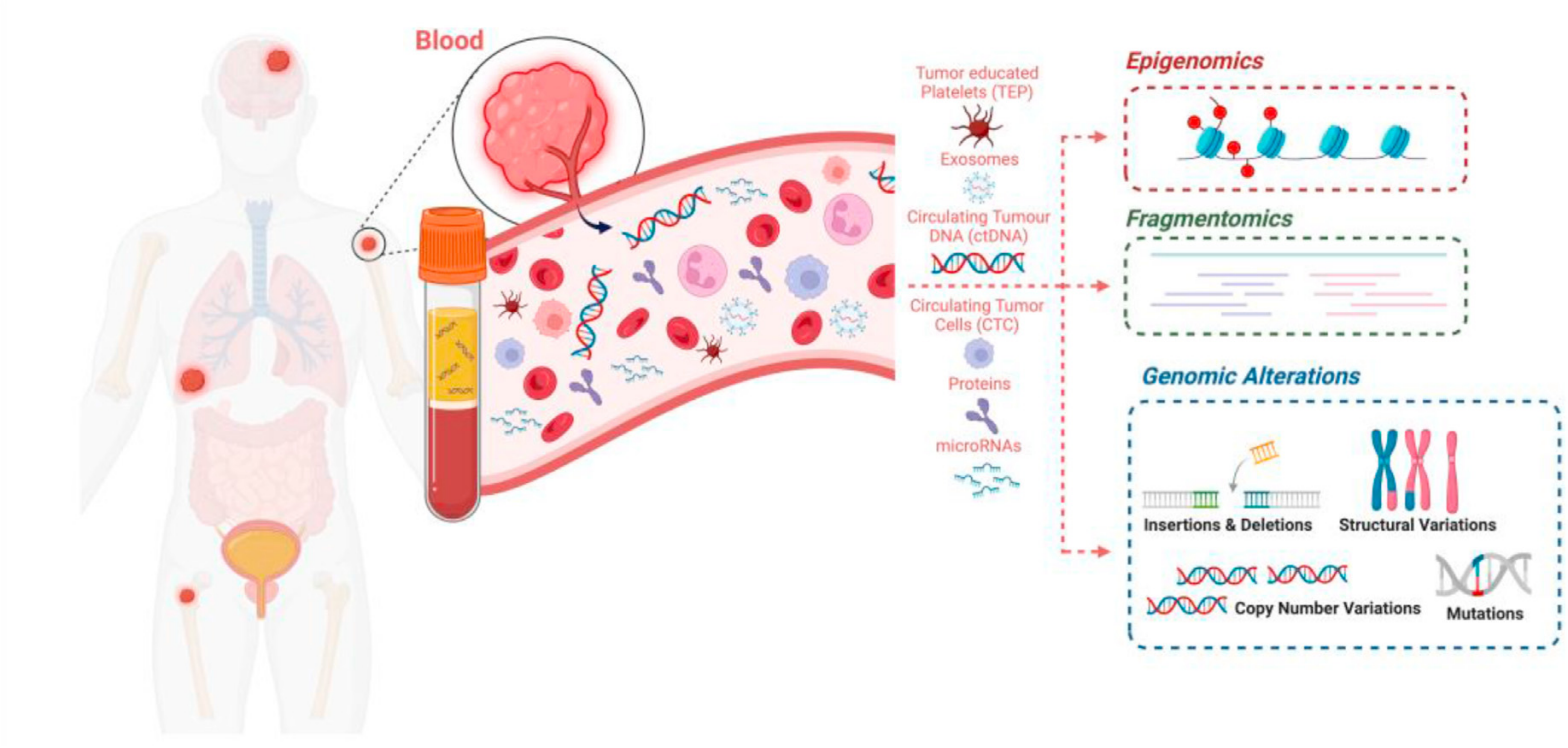
Liquid biopsy analytes.

Circulating biomarkers typically detected by liquid biopsy are circulating tumor cells (CTCs), circulating cell-free DNA (cfDNA) and ctDNA, exosomes, microRNAs (miRNA), peripheral blood circulating RNA, and tumor-educated blood platelets (TEPs) [5]; CTCs, ctDNA, and exosomes are the most frequently detected biomarkers (Fig. 1). Intact tumor cells, named CTC when in blood, can also be isolated and are shed by the tumor into the bloodstream. Compared to white blood cells, CTCs are present in very low abundance (approximately fewer than 10 cells/mL of blood in metastatic patients). This makes them challenging to detect and isolate [6].

Cell-free DNA most typically refers to small fragments of DNA released into the bloodstream from cells throughout the body, via any number of processes involved in regular cell turnover [7]. The fragments may come from both primary and metastatic sites, and vary in base pair length and alterations. Importantly, ctDNA has a very short half-life of <120 min, thus its presence is a real time reflection of tumor DNA content [8].

To maximize the reliability of molecular information obtained from a liquid biopsy, there is a need to develop a standardized workflow for the preanalytical steps. Different approaches for collecting, processing and storing blood can lead to an up to 50% variability in the amount of cfDNA extracted [9]. Each of these steps could affect the analytical outcome of certain assays resulting in different study results [10,11]. An important preanalytical goal is to achieve the highest extraction efficiency for fragments of cfDNA below 200 bp as this range contains most ctDNA fragments. Different studies have tested several extraction methods showing differences in the percentage of short cfDNA isolated depending on the kit used [12].

Evidence indicates that ctDNA can be used to detect MRD in advance clinical disease relapse, although the value of this approach in terms of improving patient outcomes requires investigation in prospective interventional trials [[13], [14], [15], [16]]. An example of such a trial is the phase II c-TRAK-TN study in patients with resectable triple-negative breast cancer (NCT03145961), MERMAID-1 (NCT04385368) study in MRD-positive patients with completely resected stage II–III NSCLC without *EGFR* mutations or *ALK* rearrangements, MERMAID-2 (NCT04642469) study in patients with stage II– III resected NSCLC without *EGFR* mutations or *ALK* rearrangements that will be regularly monitored for the presence of MRD via the analysis of ctDNA levels in plasma samples. Patients who become MRD+ during the surveillance period and have no disease recurrence visible on imaging will be randomized 1:1 to receive adjuvant therapy. Also, the Personalized Escalation of Consolidation Treatment Following Chemoradiotherapy and Immunotherapy in Stage III NSCLC in Stage III NSCLC study (NCT04585490), will assess the changes in the levels of circulating tumor DNA (ctDNA) in MRD ​+ ​subjects with stage III unresectable disease with positive DNA treated with consolidation chemotherapy and immunotherapy [[17], [18], [19]].

The data of the study Tracking Cancer Evolution Through Therapy (TRACERx) suggest that early detection of non-small-cell lung cancers (<2 cm; T1a – T1b) using ctDNA will be limited by the technical and physical constraints of detecting mutations present at a low mutant allele fraction (MAF) (<0.1%). Next-generation sequencing (NGS) platforms operating in these clinical trials must enable confident detection of mutations in plasma at a MAF of <0.1% and should therefore incorporate strategies to control for sequencing artifacts [14].

Currently, there is no standardization of the whole pre-analytical process for liquid biopsy analysis even if some progress has been made, especially for blood sample stabilization for cfDNA, cfRNA and CTC integrity. Nonetheless, most pre-analytical aspects still require deep investigation and harmonization among different research laboratories, making it difficult to compare the obtained results.

ctDNA detection for MRD-based clinical trials might be a challenge but will provide valuable information on clonal, temporal and spatial tumor behavior and give us an insight on guided therapy.

## 2 The role of ctDNA detection in minimal residual disease

Circulating tumor DNA is a multifaceted biomarker; presurgical ctDNA levels reflect relapse risk in early-stage non-small cell lung cancer (NSCLC) [[20], [21], [22]] and postoperative ctDNA detection highlights impending NSCLC recurrence [13,[21], [22], [23], [24], [25]]. Potential exists for postoperative ctDNA to guide the administration of adjuvant therapy [26,27].

MRD, also known as molecular residual disease, refers to the detection of any tumor-derived material in blood after curative-intent treatment, with the potential of achieving an earlier diagnosis of cancer recurrence, compared to standard-of-care radiologic imaging [28].

A prospective, multicenter cohort on dynamic monitoring of ctDNA in lung cancer surgery patients, collected 950 plasma samples obtained at three perioperative time points (before surgery, 3 days and 1 month after surgery) of 330 stage I-III NSCLC patients, as a part of the LUNGCA cohort [22]. Using a customized 769-gene panel, somatic mutations in tumor tissues and plasma samples were identified with next-generation sequencing and utilized for ctDNA-based MRD analysis. Preoperative ctDNA positivity was associated with lower recurrence-free survival (RFS; HR = 4.2; P < 0.001). The presence of MRD (ctDNA positivity at postoperative 3 days and/or 1 month) was a strong predictor for disease relapse (HR = 11.1; P < 0.001). ctDNA-based MRD had a higher relative contribution to relapse-free survival (RFS) prediction than all clinicopathologic variables such as the TNM stage. Furthermore, MRD-positive patients who received adjuvant therapies had improved RFS over those not receiving adjuvant therapy (HR = 0.3; P = 0.008), whereas MRD-negative patients receiving adjuvant therapies had lower RFS than their counterparts without adjuvant therapy (HR = 3.1; P < 0.001). After adjusting for clinicopathologic variables, whether receiving adjuvant therapies remained an independent factor for RFS in the MRD-positive population (P = 0.002) but not in the MRD-negative population (P = 0.283). Authors concluded that perioperative ctDNA analysis is effective in early detection of MRD and relapse risk stratification of NSCLC, and hence could benefit NSCLC patient management [22].

In the TRACERx trial, Abbosh et al. evaluated ctDNA to detect and profile residual tumor cells persisting after curative intent therapy [12]. Different relevant results were obtained. On the one hand, among the 53 patients with NSCLC relapse, some individuals, particularly those with non-adenocarcinoma histology, shed tumor DNA in blood prior to surgery. ctDNA could be detected at or before relapse in 91% (38/42) of preoperative shedders versus 64% (7/11) of nonshedders. Remarkably, the median lead time prior to relapse was 164 days in shedders versus 22 days in nonshedders [12]. Importantly, tracking MRD using the ctDNA enrichment panel heralded relapse before disease recurrence was evident on standard imaging scans. For example, 26 patients had one or more surveillance scans showing no evidence of relapse or equivocal changes. All of these individuals had a preceding blood test positive for MRD, and 24 of 26 (92%) finally developed NSCLC relapse [23].

Other relevant piece of evidence of the potential role of liquid biopsy in early NSCLC was published by Chadhuri et al. [13]. To identify MRD in early-stage lung cancer patients, CAPP-Seq was retrospectively used to search for ctDNA in 255 blood and tissue samples from 40 Stage I–III patients who underwent curative-intent treatment, as well as 54 healthy adults. ctDNA was analyzed before treatment, and the subsequent follow-up visits concomitant with radiological imaging [11]. Pre-treatment ctDNA concentration highly correlated with metabolic tumor volume and stage. All patients with detectable ctDNA in at least one post-treatment evaluation experienced recurrence [11]. Intriguingly, ctDNA was detected in the first post-treatment blood sample in 94% of patients with relapsed disease, suggesting that ctDNA is a sensible tool for detecting MRD. Furthermore, the trial demonstrated that ctDNA can also anticipate imaging-documented progression by a median of 5.2 months in 72% of patients.

Evidence suggests that liquid biopsy represents a valuable tool for ctDNA-based MRD detection and could be a prognostic and predictive biomarker in early-stage NSCLC. Prospective trials in larger series will be required to validate this approach and to evaluate whether adjuvant personalized treatment based on MRD detection can improve NSCLC survival.

## 3 Stage I-III non-small cell lung cancer patients radically treated

In stage I–III NSCLC patients who undergo curative-intent treatment, recurrence represents a challenge. The 5-year survival rate declines from 90% for stage IA1 to 41% for stage IIIA in resected lung cancer patients [29]. The Lung Adjuvant Cisplatin Evaluation (LACE) analysis demonstrated a 5-year survival benefit of 5.4% derived from adjuvant chemotherapy administration, with an overall hazard ratio (HR) of 0.89 (95% CI, 0.82 to 0.96; p = 0.005) [30].

In unresectable stage III NSCLC patients, Durvalumab administration after chemo-radiotherapy (CRT) has significantly improved PFS and OS becoming the new standard of care [31]. After radical surgery or CRT, MRD detection could allow medical oncologists to identify patients at the highest risk for recurrence, who may possibly benefit most from adjuvant therapy. In this scenario, ctDNA has emerged as an independent predictive marker of relapse in early-stage NSCLC radically treated, as reflected in the number of studies conducted to investigate its potential use (Table 1, adapted from Bertoli et al. [32]). Several studies have demonstrated the ctDNA sensitivity in MRD detection. These studies have investigated the dynamic changes of ctDNA in early-stage NSCLC patients, proving a rapid decline in ctDNA levels after surgical resection [[33], [34], [35], [36]]. Conversely, detectable plasma ctDNA after resection correlates with residual/recurrent disease.

**Table 1:** Adapted from Bertoli E. et al. *Liquid Biopsy in NSCLC: An Investigation with Multiple Clinical Implications*. Int. J. Mol. Sci. 2023 [32]. ctDNA, circulating tumor DNA; MRD, minimal residual disease; NSCLC, non–small-cell lung cancer; CAPP-Seq, Cancer Personalized Profiling by deep Sequencing; CRT, chemoradiation; cSMART, circulating single molecule amplification and resequencing technology; PORT, postoperative radiotherapy; RT, radiotherapy. Clinical sensitivity (percentage of patients who relapsed in the follow up period who were ctDNA positive) and clinical specificity (percentage of patients who did not relapse in the follow up period who were ctDNA negative) were calculated for the first follow up sample after completing definitive therapy (ctDNA MRD Landmark). ∗ In the paper, a positive predictive value of landmark has been reported for relapse of 93% and a negative predictive value of landmark for relapse of 68%.

Study	Number of Patients	Clinical Stage	ctDNA Methodology	Mutations Monitored	Treatment	ctDNA MRD Landmark	
						Sensitivity	Specificity
Chaudhuri et al. [11]	37	IB-IIIB	CAPP-Seq	Multiple	CRT or RT and/or surgery ± chemotherapy	94%	100%
Chen et al. [18]	25	IIB-IIIB	cSMART	Multiple	Surgery ± chemotherapy	44%	88%
Moding et al. [19]	12	IIB-IIIB	CAPP-Seq	Multiple	CRT	100%	100%
Abbosh et al. [12]	108	IA-III	Anchored-multiplex PCR (AMP	Multiple	Surgery ± chemotherapy	49%	N.E.∗

Currently, CT-scan imaging every 8–12 weeks comprises the standard follow-up in these patients after definitive treatment. Liquid biopsy is minimally invasive and may anticipate radiological disease relapse. In this setting, liquid biopsy may change adjuvant treatment paradigm, although further prospective studies in larger series of patients are needed.

Among the whole population of early-stage NSCLC patients, a subgroup of individuals in whom ctDNA monitoring may be more easily performed and may result clearly more informative is the subpopulation of NSCLC patients with actionable mutations. For example, a recent study has evaluated the feasibility and efficacy of longitudinal monitoring of ctDNA as a valuable biomarker for early detection of MRD and to provide identification of the group at high risk for recurrence in resected stages I to IIIA *EGFR* mutation positive (*EGFR*-M+) NSCLC [37]. In addition to MRD analysis, to validate variants in plasma samples, droplet digital PCR (ddPCR) was performed. The authors showed that with longitudinal monitoring of ctDNA, MRD was detected before radiological recurrence in 69% of patients with exon 19 deletion and in 20% with L858R mutation in *EGFR*. Moreover, ddPCR captured *EGFR* mutations in ctDNA before radiologic relapse in 54% and 11% of the cases with *EGFR* del19 and L858, respectively [37].

## 4 Material and methods

In this review, we present four clinical cases of patients with NSCLC, localized or locally advanced disease, and *EGFR* mutation. Of all these cases, 3 were stage I and were treated with surgery with curative intent, while one of them (the second) was a patient with an unresectable stage III *EGFR* mutated NSLC and was treated with concurrent chemoradiotherapy with radical intent. Periodic radiological assessment and liquid biopsy analyses were carried out for follow-up. These four patients do not truly reflect a thorough analysis of MRD after radical-intent treatment, but the detection of previously tumor tissue-detected *EGFR* mutations in cfDNA of those patients using an NGS test or a Cobas® targeted approach. The post radical treatment early detection of an *EGFR* mutation in a ctDNA test is feasible and accessible to most clinical practices and therefore represents a more practical clinical use approach.

Plasma samples from peripheral blood were collected and cfDNA was isolated using two different methods.

1. The technique used in the first three patients who will be described below was Oncomine Pan-Cancer Cell-Free Assay (Thermo Fisher scientific, Waltham, MA, USA) to determine the *EGFR* status in plasma cell-free DNA (cfDNA). This approach can identify single-nucleotide variants (SNVs), indels, copy number variations (CNVs), and RNA fusions within 52 genes.
2. In the last patient, the commercial kit Cobas® *EGFR* Mutation Test v2 CE-IVD (Roche Diagnostics, SL) was used. The cobas® *EGFR* Mutation Test v2 uses real-time PCR technology to detect and identify 42 mutations in exons 18, 19, 20 and 21 in the *EGFR* gene using plasma samples. The cobas® *EGFR* Mutation Test v2 included the Semi-Quantitative Index (SQI). The SQI is a measure of the amount of mutant cfDNA in a sample and can be used to measure differences in mutation load over time. An increase or decrease in the SQI value indicates a respective change in the amount of corresponding target mutation in an individual patient.

### 4.1 Plasma preparation

For the Oncomine Pan-Cancer Cell-Free Assay, blood samples were drawn and collected into 3.8% sodium citrate (EDTA) sterile tubes. Plasma processing was done within 4 h from extraction and preserved at −80°C. Samples were tempered to 4°C to carry out a new centrifugation process to obtain at least 6 ml (maximum volume that should be analyzed).

For the Cobas® *EGFR* Mutation Test v2 CE-IVD, blood sample was drawn and collected into a Cell-Free DNA BCT. This tube stabilizes cell-free DNA for up to 14 days at 6 °C–37 °C. Plasma processing was separated across a two centrifugation process and preserved −80°C. 2 ml of sample was use for cell free DNA extraction.

### 4.2 Nucleic acid extraction. Cell-free Total Nucleic Acid (cfTNA)

For the Oncomine Pan-Cancer Cell-Free Assay, proteinase k was added to the sample and high concentration of DNA was achieved through a magnetic beads binding process (MagMax cell-free Total Nucleic Acid Magnetic Beads). DNA was quantified in QUBIT 3.0 (Qubit dsDNA HS), obtaining at least 10 ng (10–100 ng) of concentrated cfTNA.

The Cobas® cfDNA Sample Preparation Kit is used for manual sample preparation to isolate circulating cfDNA from plasma samples based on nucleic acid binding to glass fibers, for use in combination with our Cobas® oncology assays, including the Cobas® *EGFR* Mutation Test v2. Cell Free DNA was quantified in bioanalyzer Agilent TapeStation system using a High Sensitivity DNA ScreenTape Analysis. Agilent TapeStation system is an automated electrophoresis solution for the sample quality control of cfDNA and provides accurate sizing and quantification of cfDNA, as well as DNA quality assessment with the objective percentage of cfDNA quality metric.

### 4.3 Library preparation, sequencing, and variant calling

Transcription of RNA to DNA was carried out using SuperScript VILO Master Mix to obtain as much information as possible from the sample and proceeded to the amplification and purification of the "target" regions of the panel. The Oncomine Pan-Cancer Cell-Free Assay was used for library preparation, in which DNA fragments were uniquely tagged. Libraries were loaded using an Ion 540 kit using Ion Chef (Thermo Fisher Scientific, Waltham, MA, USA).

After quantification with Qubit (Thermo Fisher) for total circulating free nucleic acids extracted with the MagMax Cell-free Total Nucleic Acid kit (Thermo Fisher), sequencing, identification and annotation of variants was carried out based on the nomenclature recommendations of the Human Genome Variation Society (HGVS). Regions of interest were enriched with the Oncomine ™ PanCancer-Cell-Free Assay kit (Thermo Fisher) using between 10 and 100 ng of cfTNA. Subsequently, 4 NGS libraries are sequenced on an Ion 540 ™ chip using the IonS5 ™ System (Thermo Fisher) allowing the identification of different types of variants in the following genes.

- Point mutations (SNVs/indels): *AKT1, ALK, APC, AR, ARAF, BRAF, CHEK2, CTNNB1, DDR2, EGFR, ERBB2, ERBB3, ESR1, FBXW7, FGFR1, FGFR2, FGFR3, FGFR4, GLT3, GNA11, GNAQ, GNAS, HRAS, IDH1, IDH2, KIT, KRAS, MAP2K1, MAP2K2, MET, MTOR, NRAS, NTRK1, NTRK3, PDGFRA, PI3CA, PTEN, RAF1, RET, ROS, SF3B1, SMAD4, SMO*, and *TP53*
- Amplifications (CNVs) in *CCND1, CCND2, CCND4, CDK4, CDK6, EGFR, ERBB2, FGFR1, FGFR2, FGFR3, MET* and *MYC*
- Rearrangements (translocations/fusions) in *ALK, BRAF, ERG, ETV1, FGFR1, FGFR2, FGFR3, MET, NTRK1, NTRK3, RET* and *ROS.*

#### 4.3.1 Reading depths ranged between 6008X and 12362X

For manual sample preparation, plasma specimens are processed using the Cobas® cfDNA Sample Preparation Kit. The Cobas® EGFR Test is based on two major processes [1]: manual sample preparation to obtain DNA from plasma; and [2] PCR amplification and detection of target DNA using complementary primer pairs and oligonucleotide probes labeled with fluorescent dyes. The Cobas® *EGFR* Test is designed to detect the following mutations: Exon 18: G719X (G719A, G719C, and G719S); Exon 19: deletions and complex mutations (defined as the combination of a deletion and an insertion); Exon 20: S768I, T790 M, and insertions; Exon 21: L858R and L861Q. The Cobas z 480 analyzer is used for automated amplification and detection.

### 4.4 Data analysis

For the Oncomine Pan-Cancer Cell-Free Assay, after sequencing data was generated, the Torrent Suite software (5.16.0) carried out a secondary analysis. This included quality control (QC), identification and annotation of variants (Workflow of Pan-Cancer 5.10) in the Ion Reporter Local (5.10.1.0) from Thermo Fisher. Following tertiary analysis, variants that met the following criteria, which were near the detection limit of the test, were reflected in the final report.

- Point variants (SNV/INDELS) that are detected in two or more molecular families
- Fusions that are detected in more than 2 families, if the sample passes the QC value of the RNA library and
- Amplifications (CNVs) with p value > 10 -5 and ratio >1.15 if the sample passes the QC value of the DNA library with MAPD <0.4.

For the Cobas® EGFR Mutation Test v2 CE-IVD, mutation detection is achieved through PCR analysis with the Cobas z 480 analyzer. A mutant control and negative control are included in each run to confirm the validity of the run.

### 4.5 Sample limitations

#### 4.5.1 Oncomine Pan-Cancer Cell-Free Assay

Some mutations cannot be identified by this test. There are characteristic regions of the genome that make it impossible to accurately determine changes in the sequence. Homopolymeric regions cannot be accurately sequenced. The presence of rare variants in the DNA sequence could make it complex to obtain enough amplified sequences, adding difficulty to obtain a reliable result for this specific region of the genome. Variants in circulating noncoding DNA, pseudogenes, trinucleotide repetitive expansions, or epigenetic alterations are not detected. There may be real variants in the sample that cannot be reported because they are below the established sensitivity threshold. All single number variants (SNVs) are considered positive even if they are detected in a single molecular family. Copy number alterations (CNVs) are not considered if the DNA library does not meet the minimum quality requirements. The RNA library is valid and carrying the informative rearrangements as long as the expression controls are detected in more than 2 copies.

#### 4.5.2 Cobas® EGFR test

The detection of a mutation is dependent on the number of copies present in the sample and may be affected by sample integrity, amount of isolated DNA, and the presence of interfering substances. The presence of PCR inhibitors may cause false negative or invalid results. Samples tested outside the linear range of the assay may generate false results. Samples with results reported as “No Mutation Detected” may harbor *EGFR* mutations not detected by the assay. The Cobas® EGFR Test shows cross-reactivity (results of “Mutation Detected”) to the exon 19 L747S mutation, a rare acquired mutation that may confer resistance to TKI treatment.

## 5 Cases presentation

Case 1 We present the case of a 67-year-old non-smoker patient at the time of diagnosis. Personal history includes dyslipidemia, elevated baseline glycemia, and prostate adenocarcinoma treated with radical prostatectomy in March 2006, with no evidence of recurrence.

Our patient was enrolled in an early lung cancer diagnosis program due to first and second-degree family history. In 2010, bilateral pulmonary nodules of few millimeters were detected in a CT scan. Follow-up continued until 2018. At that point, after observing the growth of one of the nodules (15 × 13 mm), an extended atypical resection was performed using video-assisted thoracoscopy, with a diagnosis of atypical adenomatous hyperplasia.

The March 2021 CT scan revealed significant growth of a peribronchovascular pulmonary nodule measuring 16 × 12 mm in the right lower lobe (doubling time of 248 days) with a solid central component and a ground glass peripheral component. PET-CT confirmed the presence of a high-uptake nodule in the right lower lobe (SUVmax of 4.9), suggesting malignant disease without evidence of distant metastasis.

The patient was referred to the thoracic surgery clinic for evaluation, and surgical resection was decided in the multidisciplinary board. In April 2021, the patient underwent surgery involving right lower lobectomy, hilar lymphadenectomy, and robotic mediastinal lymphadenectomy.

Pathological diagnosis indicated moderately differentiated lung adenocarcinoma with a solid pattern in 80% and micropapillary in 20% of the tumor, isolated lepidic foci without lymphatic or vascular invasion, and surgical margins free of tumor. Eleven lymph nodes were isolated, all unaffected (pT1bN0).

Immunohistochemical study revealed a deletion in exon 19 of EGFR with a PDL-1 expression of 5%. Following clinical guidelines, the patient did not receive adjuvant treatment. Currently, the patient undergoes regular monitoring with CT imaging tests, and a liquid biopsy (Oncomine Pan-Cancer Cell-Free Assay) is performed every three months to determine circulating ctDNA.

There has been no evidence of recurrence, with liquid biopsies conducted until October 2023 showing negative results. Additionally, imaging scans do not reveal any suspicious lesions to date.

Case 2 Below, we present the case of a 77-year-old man with not known drug allergies, medical history of arterial hypertension and dyslipidemia under treatment, subclinical hypothyroidism not requiring replacement therapy, spondyloarthrosis, cholelithiasis, and hepatic steatosis. He was previously diagnosed with a depressive disorder treated with lithium until 2013 and was an ex-smoker for 58 years. There was not known family history of cancer.

In late 2020, he sought medical attention due to the presence of a non-painful parotid nodule. On examination, it was identified as a mobile subcutaneous formation located in the anterior cervical region. He was seen by our otorhinolaryngology service in December 2020, where a fine needle biopsy of the parotid lesion was performed, yielding a pathological result of Warthin's tumor.

To complete the study, a cervical thoracoabdominal CT scan was requested, revealing a suspicious-looking nodule in the upper segment of the right lower lobe with spiculated edges, measuring 13 × 12 mm, along with a subcentimeter satellite nodule of 7 × 6 mm, and small subpleural nodules in the middle lobe of the right lower lobe, with a maximum size of 8 × 7 mm, without significant lymph nodes.

On December 21, 2020, a right parotid nodulectomy was performed with excision of the cervical lipoma, resulting in a definitive diagnosis of Warthin's tumor and subcutaneous lipoma.

He was referred to the Pulmonology department, where a positron emission tomography-computed tomography (PET-CT) scan was conducted. The PET-CT showed focal uptake in the right lower lobe consistent with a pulmonary nodule, with lymph node uptake in the right pulmonary hilum, as well as in the right paratracheal, precaval, subcarinal prevascular, aortopulmonary, and right supraclavicular window (stage IIIB).

Cerebral magnetic resonance imaging on December 2020 revealed no findings suggestive of malignancy.

On January 4, 2021, an ultrasound bronchoscopy was performed, identifying lymph nodes in the right paratracheal region. Biopsies were carried out, resulting in a diagnosis of poorly differentiated lung adenocarcinoma with a high expression of PDL-1 (60%) and L858R mutation in exon 21 of the EGFR gene (Oncomine Pan-Cancer Cell-Free Assay).

Due to the unresectable nature of the disease, chemo-radiotherapy treatment with radical intent was recommended. Considering the mutation's sensitivity response to the disease, initial completion of radical treatment with maintenance immunotherapy in case of radiological stability or improvement was not supported due to its low efficacy. The patient accepted the therapeutic recommendation, receiving first intravenous cycle of induction chemotherapy on January 18, 2021. From March 4 to April 20, 2021, concurrent chemoradiotherapy treatment was administered with a total dose of 67.32 Gy using carboplatin and weekly paclitaxel for 4 weeks. The treatment tolerance was good, with no significant toxicity. On May 27, 2021, the patient attended the first follow-up, and a liquid biopsy was performed, showing no presence of the L858R mutation (Oncomine Pan-Cancer Cell-Free Assay). Regular check-ups were recommended. On August 31, 2021, a liquid biopsy determination was performed again, revealing the presence of the mutation susceptible to follow-up in the liquid biopsy p.(L858R) in the EGFR gene (NM_005228.4).

**Fig. 2:**
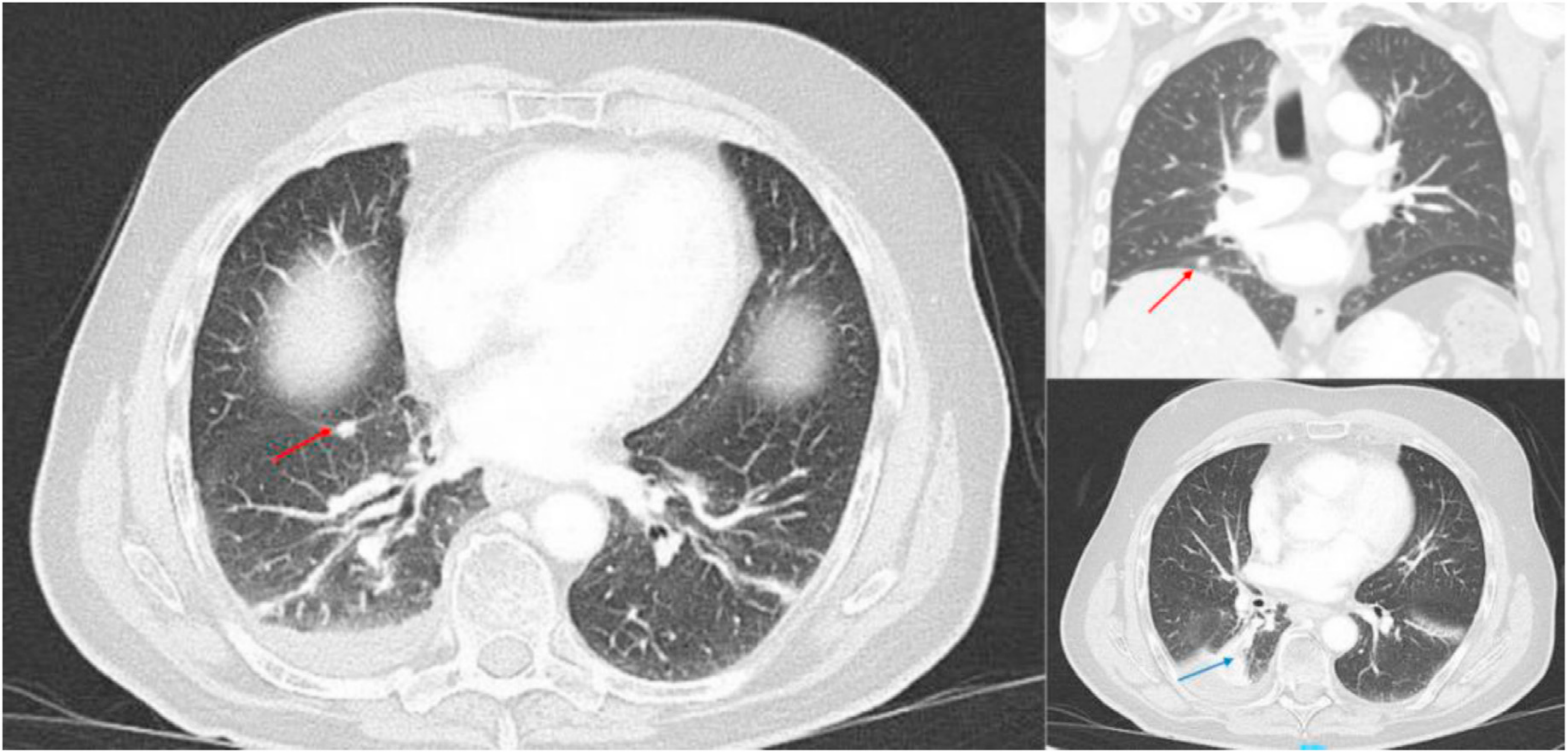
Case 2. Thorax and abdominal CT scan (August 31, 2021). Signs of radiation pneumonitis primarily affecting the upper segment of the right inferior lobe (blue arrow). Nodule of 6.8 mm in the same lobe (red arrow). No other pathological findings.

Based on these findings, a thoracic-abdominal CT was requested, detecting the appearance of a new nodule of 6.8 mm in the lower right lobe (Fig. 2). A study with cerebral magnetic resonance ruled out metastatic involvement. It was recommended to conduct a PET-CT 4 weeks later.

**Fig. 3:**
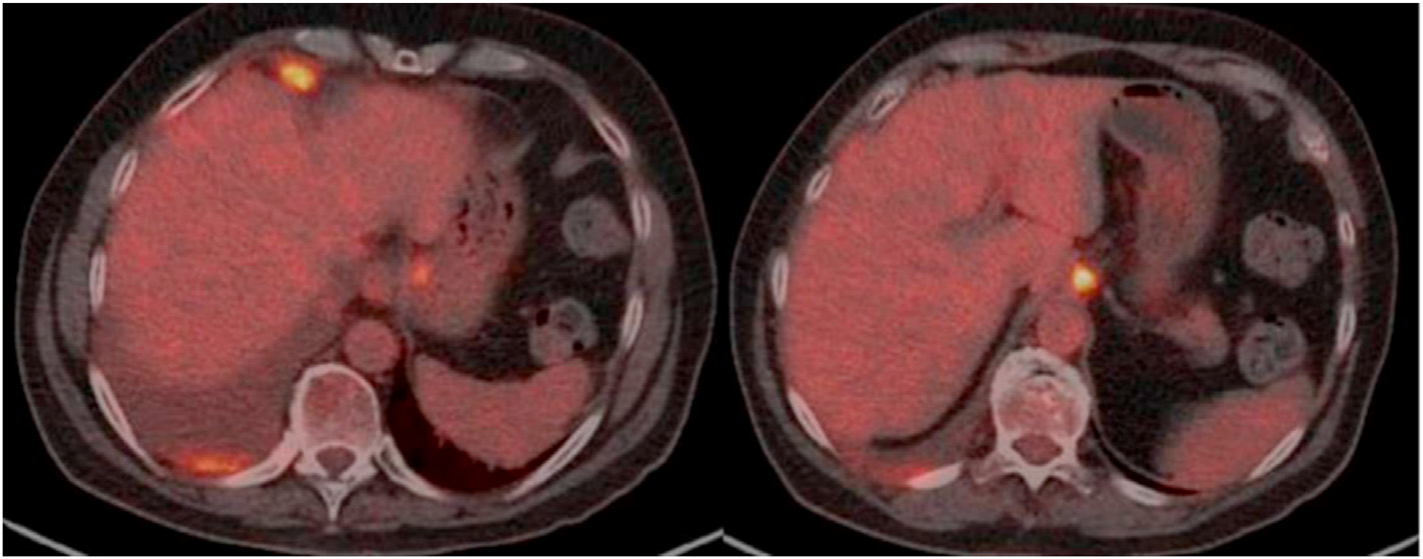
Case 2. PET/CT scan (September 28, 2021). ^18^FDG uptake in the apical, posterior and lateral right pleura with ipsilateral pleural effusion. Hypermetabolic new lesion in the right cardiophrenic space, and lymph nodes with high 18-FDG uptake in the area of the gastrohepatic ligament and pre-aortic.

PET-CT showed right pleural and retroperitoneal progression, as well as progression in the right pleura, diaphragm, and right supraclavicular lymph nodes (Fig. 3). None of those findings were previously identified by the CT scan performed.

After confirming disease progression, initiating treatment with osimertinib at a daily dose of 80 mg was proposed. Initially, treatment tolerance was good. Unfortunately, the patient experienced clinical deterioration after three months of treatment, requiring hospitalization, and ultimately succumbed to disease progression.

Case 3 We present the case of an 82-year-old woman at the time of diagnosis, a non-smoker with no relevant past medical history. In December 2016, she experienced pain in the right hemithorax accompanied by fever. She had no cough or expectoration. At that time, she consulted her primary care physician, who performed a chest X-ray revealing an infiltrate in the right middle lobe, leading to a provisional diagnosis of possible pneumonia. Antibiotic treatment was initiated, resulting in the resolution of fever.

During a follow-up radiological examination in March 2017, no changes were observed in the image of the middle lobe. Subsequently, a chest and abdominal computed tomography (CT) scan confirmed the presence of a 3.3 cm right parahilar mass. No hilar or mediastinal adenopathies were evident.

On April 25, 2017, she presented to our center, where a PET-CT scan confirmed a mass in the right middle lobe with increased uptake, reaching a maximum of 9.4. Additionally, an area of distal pneumonitis was noted. Increased uptake at the right hilar level with a maximum SUV of 3.2 raised suspicion of lymph node infiltration.

An MRI ruled out the presence of brain disease. A bronchoscopy with biopsy was performed, and the diagnosis was consistent with adenocarcinoma.

In May 2017, a regulated median lobectomy was carried out, accompanied by hilar and mediastinal lymphadenectomy, resulting in a diagnosis of moderately differentiated adenocarcinoma. Surgical margins were free of disease. TTF1 was negative, with no expression of *p40*, *ALK*, or *ROS-1*, and PDL-1 expression was observed in 1% of the cells. No lymph node infiltration was observed in the nodes obtained during surgery. Molecular analysis through NGS in the tumor tissue sample revealed the presence of an activating EGFR mutation, specifically L858R.

With a diagnosis of moderately differentiated lung adenocarcinoma stage pT2aN0M0 (IB), she was referred to the Medical Oncology department. After assessing the advantages and disadvantages, no adjuvant treatment was given. Instead, a liquid biopsy study was conducted to determine the presence of an activating EGFR mutation (Oncomine Pan-Cancer Cell-Free Assay), with no evidence of this alteration after surgical treatment.

Since then, the patient has been undergoing regular follow-ups with no recurrence of the EGFR mutation in subsequent liquid biopsy examinations. Additionally, there has been no evidence of relapse in imaging tests conducted to date.

Case 4 Finally, we present the case of a 77-year-old patient at the time of diagnosis. A former smoker for 20 years, averaging one pack per week, with a medical history of unstable angina involving 2-vessel disease and incomplete revascularization in May 2021. The patient had undergone several surgeries for degenerative pathology.

An incidental finding of a lung node in the right upper lobe was made in the context of a respiratory tract infection. Further investigation through a thoracic and abdominal CT scan revealed a spiculated nodule in the right upper lobe measuring 15 mm, raising suspicion of malignancy. PET-CT ruled out distant lesions but confirmed hypermetabolism of the identified nodule (SUVmax 1.6). A core needle biopsy was performed, confirming malignancy as pulmonary adenocarcinoma with a lepidic pattern.

Following appropriate respiratory functional tests and cardiology assessment, on November 29, 2022, a right upper lobectomy with staging lymphadenectomy was conducted. The pathological report confirmed a pT1aN0 lung adenocarcinoma, PD-L1 <1%, and an EGFR mutation (p*.*L858R exon 21) was identified by Cobas® test in tissue sample. The case was presented in the multidisciplinary tumor board, and given the IA1 stage, a decision for surveillance was made.

Liquid biopsy (Cobas® EGFR Test) at follow-up appointments (2/2023, 6/2023, and 12/2023) have not detected the mutation in the blood. These findings correlated with CT studies that showed no signs of tumor recurrence.

## 6 Discussion

### 6.1 Pre-analytical and analytical values

Within the preanalytical phase, the aspect of the tumor being characterized as well as the most appropriate sample to be collected are key decisions to be made prior liquid biopsy testing implementation.

First, liquid biopsy is a general concept that does not necessarily define the aspect of the tumor being characterized. The tumor during its evolutionary process, suffers from apoptosis, for example, so components of those tumor cells can be characterized starting from DNA but also RNA, proteins or even lipids. As a note, some tumor cells do achieve the ability to migrate so we can also study DNA, RNA, proteins or even lipids of those circulating tumor cells. Therefore, the first decision that one needs to make is what aspect of the tumor will be characterized by liquid biopsy.

However, even if once decides, for example, cell free DNA, the most appropriate “non-invasive” sample that could contain a higher proportion of tumor cell free DNA needs to be chosen. Indeed, whole blood-derived plasma is the most common non-invasive sample being clinically studied; however, if the tumor is within the biliary tract, bile may be a more suited sample to be tested; while if the tumor is in the brain area, cerebrospinal fluid. Even if plasma is chosen to be the source of cell-free DNA to be studied, the time between the blood draw and plasma isolation and freezing at −20C or even −80C is one of the most important, if it is not the most important, step. Cell free DNA and RNA can easily be degraded during that “short” period of time.

Finally, cell free DNA needs to be isolated from each of the plasma samples. There is a diversity of methods that allow cfDNA to be isolated from plasma samples including both manual and automatic protocols. One way or the other, each laboratory performing cfDNA isolation needs to make sure to implement control quality steps to understand the amount and quality (i.e., degradation level) of the isolated cfDNA.

The pre-analytical steps of ctDNA mutation testing can be impacted by many factors: 1) timing of blood draw; 2) storage of cell-free plasma; 3) sampling tubes considerations; 4) plasma and cfDNA-isolation protocols; and 5) quality assessment methods for cfDNA integrity [38].

In addition, the analytical phase has several limitations worth to be mentioned. Once we have isolated cfDNA, different analytical methods can be used to molecularly characterize it; for example, quantitative PCR (qPCR) or digital PCR (dPCR). Both methods have some similarities, but also important differences to be considered. Both can identify SNV or even small indels with low variant allele frequencies (VAFs); however, qPCR measures the accumulation of DNA during one PCR reaction while dPCR measures DNA accumulation in thousands of individual reactions/partitions; for example, 200,000. Therefore, digital PCR is a more precise approach to sensitive nucleic acid detection and quantification without the need of standard curve for quantification (as qPCR does). However, they both have a common limitation, you need to know which alternative variant you are looking for. On the other hand, as we have shown before, we do have an alternative method to assess the presence or absence of variants within both DNA as well as RNA within a wider genomic region (and without knowing which alternative allele we are looking for) and with the same sensitivity and accuracy that dPCR has. This alternative method is called next-generation sequencing (NGS). NGS can indeed sequence the whole genome within an isolated cfDNA samples: however, in order to achieve the sensitivity that is required in liquid biopsy (due to TF), the cost of using NGS for whole genome sequencing is too elevated; therefore, targeted-based NGS sequencing is currently used in clinical settings as we have shown in the previous section.

In the COBAS***®*** *EGFR* test case, 1) When we receive streck-peripheral blood tubes, the cell-free plasma is isolated in the same day or as maximum 3 days later at room temperature; 2) once the cell-free plasma is processed, is stored at −80°C; 3) we use a collection tube that preserves cfDNA at room temperature (the International Association for the Study of Lung Cancer-IASLC recommends 3 days as a maximum); 4) We process 2 ml of plasma to obtain cfDNA keeping a manufacture standard protocol by Roche®. This is a manual kit specifically designed for cfDNA isolation from plasma based on nucleic acid binding to glass fibers; 5) We check the isolated cfDNA integrity with High Sensitivity D1000 ScreenTape by Tape Station bioanalyzer (Agilent), from which fragment sizes and calibrated concentration (pg/ul) are obtained. A cfDNA extraction would be optimal for analysis when cfDNA fragment sizes are > to 200 bp [39].

In our clinical case number 4, we used a PCR-based method (COBAS***®*** *EGFR* test v2 (Roche®)), that was approved by the FDA in 2016 as the first test for the identification of NSCLC patients harboring *EGFR* mutations in cfDNA from plasma for TKI treatment selection. This test consists in a real-time PCR that interrogates 42 mutations located in exons 18, 19, 20, and 21 of the *EGFR* gene and provides a semiquantitative index (SQI) for the *EGFR* mutation, which reflects the trend for the proportion of mutated versus wild-type copies of the *EGFR* gene in the cfDNA. However, the COBAS® *EGFR* Test is not approved as a quantitative test. The COBAS® *EGFR* tests include a positive and negative control, as well as an internal control for each sample; it has a detection limit of around 0.1–1% of the variant allele fraction (VAF) [40].

To validate our pre-analytical and analytical procedures, we participate in annual external quality assessments (EQA) for cfDNA testing in lung cancer, as a requirement to guarantee the proficiency of our test.

### 6.2 Methodological considerations, disadvantages, and limitations for disease follow-up

The persistence of ctDNA after the surgical resection of a primary tumor may indicate either disease persistence or recurrence, depending on the time point. In this context, the specific timing of blood extraction after surgery is a key consideration for minimal residual disease (MRD) and can assist in monitoring treatment response, potentially altering cancer management [41].

Prior detection of mutations in tumor tissue enables targeted monitoring of a larger number of variants, facilitating more reliable detection during follow-up. The exclusive use of liquid biopsy poses the risk of analyzing many regions unlikely to contain a variant of interest, thereby increasing the number of false-positive results. Tumor-based approaches may help reduce the rate of false positives. To mitigate this loss of specificity, some experts recommend identifying at least two matching variants in both tumor and plasma [42].

Frequently, ctDNA concentrations are low (0.1% of cfDNA) [43], depending on several factors (tumor location, stage, non-shedding tumors or cases of brain metastases, other non-tumor neoplasms), making necessary methods with high analytical sensitivity for successful ctDNA detection. Detection methods include digital polymerase chain reaction, multiplex polymerase chain reaction–based next-generation sequencing (NGS), and hybrid capture–based NGS [44]. Other disadvantages are the low copy number of mutant alleles due to the median half-life of ctDNA, that ranges from 15 min to a few hours; a higher risk of producing non-informative results (e.g. artifacts, mutations that are not currently actionable); and the increase of overall costs as consequence of testing serial samples [[38], [39], [45]].

Currently, consensus is needed on which methodologies can provide the most accurate information. While PCR-based approaches to analyze well-characterized mutations in clinically relevant genes such as *EGFR* are most commonly used, broader NGS gene panels could be beneficial in specific clinical scenarios [46].

A significant challenge to maintaining the specificity of ctDNA testing is confounding by clonal hematopoiesis of indeterminate potential (CHIP). CHIP modifications arise from hematopoietic progenitor cells. Some studies have shown that up to 14% of patients with early-stage lung cancer and up to 25% of patients with advanced solid tumors harbor CHIP mutations [20,[47], [48], [49]].

False positives constitute an important limitation when making decisions, as they could expose the patient to tests or treatments that affect their quality of life, causing toxicities and doubtful benefits.

In 2018, a multidisciplinary panel concluded that the regular use of liquid biopsy provided a potential improvement in patient care and follow-up. Even so, as we have discussed throughout this review, the determination of ctDNA has some limitations and there is the possibility of obtaining uninformative or artefactual results. In addition, it is necessary to establish in a specific way in which cases it could be useful since patients with non-shedding tumors or brain tumors may have very low levels of ctDNA [50].

### 6.3 Advantages

It is noteworthy that there are high concordance rates between determinations of mutations in tumor biopsy and liquid biopsy, which are around 91–95% [51].

Although tissue biopsy is the gold-standard diagnostic method used to obtain or to confirm the diagnosis of cancer, to define histological subtype, or to identify actionable targets, molecular profiling from circulating tumor DNA (ctDNA) in patients with non-small-cell lung cancer (NSCLC) has several advantages, including: its minimally invasive nature; a continuous monitoring of genetic alterations during patient outcome; the best reflection of intratumor and intertumoral heterogeneity; and, can be analyzed from bio-banked biofluids [38].

Another benefit that liquid biopsy surveillance could provide for early detection of relapse is key in disease management. Therapy directed against minimal residual disease is already a reality of treatment for hematological malignancies. A recent example is the early FDA approval of blinatumomab for patients with B-cell leukemia who presented MRD after initial chemotherapy [52]. Trials that take circulating ctDNA as a reference to establish a therapeutic approach are currently in very early stages. It is still necessary to establish to what extent it is a determining diagnostic parameter.

### 6.4 Future perspectives: liquid biopsy in the neoadjuvant setting

In recent years, some neoadjuvant studies for resectable non-small cell lung cancer have incorporated liquid biopsy to detect the clearance of ctDNA after neoadjuvant treatment with chemotherapy and immunotherapy [53,54]. In these studies, the patient undergoes a liquid biopsy prior to the administration of each systemic cycle, as well as another one before the surgical procedure.

In one of these phase III trials, a significant correlation has been observed between patients achieving complete pathological response or major pathological response and ctDNA clearance [54], highlighting its potential impact on survival. Although we need more information regarding liquid biopsy and response to neoadjuvant treatment, these results may help guide us in deciding the most appropriate course of action after systemic treatment. This could involve tailoring subsequent approaches to be more or less aggressive based on these findings.

## 7 Conclusion

Liquid biopsy is beginning to position itself as a very useful tool in the management, among other tumors, of non-small cell lung cancer, including early diagnosis, identification of minimal residual disease (MRD), detection of predictive and prognostic markers, assessment of resistance mechanisms, and monitoring treatment response.

The cases presented underscore the clinical relevance of liquid biopsy in the context of *EGFR* mutant NSCLC patients who received standard curative treatment. The integration of liquid biopsy, utilizing techniques such as the Oncomine Pan-Cancer Cell-Free Assay and Cobas® EGFR Mutation Test, provided valuable insights into the genetic landscape of these patients.

There is still a path to be travelled; the limitations of the technique in detecting minimal residual disease include primarily obtaining false positives, confusion with clonal hematopoiesis of indeterminate potential, and the need for consensus on the most accurate detection methodologies. Despite these challenges, we highlight the high concordance rates between liquid biopsy and tumor biopsy results.

New applications of liquid biopsy are looming in early-stage lung cancer: specifically, those related to the response to neoadjuvant treatment. Early trials incorporating liquid biopsy in neoadjuvant studies indicate promising correlations between ctDNA clearance and pathological response, paving the way for potential adjustments in post-systemic treatment approaches.

In essence, this review advocates for the continued exploration and integration of liquid biopsy in routine clinical practice for NSCLC patients. While challenges persist, the potential impact on personalized treatment strategies, early detection of recurrence, and improved patient outcomes make liquid biopsy a valuable and evolving tool in oncology.

## Ethical responsibilities

The authors declare that no experiments have been conducted on humans or animals for this research. Likewise, they declare that patient data is not included and informed consent has been obtained from them.

## Declaration of competing interest

The authors declare that they have no known competing financial interests or personal relationships that could have appeared to influence the work reported in this paper.
